# Changes in multidrug-resistant Enterobacteriaceae features among patients in the emergency room during the COVID-19 Omicron variant pandemic

**DOI:** 10.1371/journal.pone.0355725

**Published:** 2026-08-17

**Authors:** Li-Feng Hung, Ping-Xuan Guo, Chia-Yi Hou, Yus-Han Kuo, I-Jung Feng

**Affiliations:** 1 Department of Clinical Pathology, Chi Mei Hospital, Liouying, Tainan, Taiwan; 2 Institute of Precision Medicine, National Sun Yat-Sen University, Kaohsiung, Taiwan; Children’s National Hospital, George Washington University, UNITED STATES OF AMERICA

## Abstract

The purpose of this study was to investigate stage-specific changes in risk factors and resistance patterns for multidrug-resistant Enterobacteriaceae (MDRE) bloodstream infections in emergency department (ED) patients during the COVID-19 Omicron wave. This retrospective cohort study focused on the ED of a tertiary care medical center in Taiwan from January 2022 to January 2023. Participants were adult ED patients (≥20 years) with positive blood cultures for Enterobacteriaceae and complete antimicrobial susceptibility testing (AST) data. Pandemic phases were defined using national surveillance data: Stage I (pre-peak), Stage II (epidemic peak), Stage III (post-peak). An exposome-wide association study identified stage-specific clinical and laboratory variables associated with MDRE infection. Resistance trends for key bacteria–antibiotic pairs were analyzed across stages. Of 1,269 screened ED patients, 816 met inclusion criteria; 287 (35.2%) had MDRE. In Stage I, age 75–85 years (OR 2.48, 95% CI 1.16–5.43) and ≥85 years (OR 2.29, 95% CI 1.07–5.00), along with RBC, Hb, and Hct, were associated with MDRE. In Stage II, platelet count (OR 1.00, 95% CI 1.00–1.01) and ALT (OR 1.00, 95% CI 0.99–1.00) were associated. In Stage III, WBC (OR 1.04, 95% CI 1.00–1.08), segmented neutrophils (OR 1.03, 95% CI 1.00–1.05), Hb, and Hct were associated. *Klebsiella pneumoniae* maintained 100% ampicillin resistance with rising ciprofloxacin resistance, while *Escherichia coli* showed declining resistance to multiple agents. In conclusion, MDRE-associated variables and resistance patterns varied across pandemic stages, supporting the need for dynamic, stage-adapted risk assessment and antimicrobial stewardship strategies in acute care settings.

## Introduction

Multidrug-resistant Enterobacteriaceae (MDRE) infections are a growing global public health concern, particularly in acute care settings. The World Health Organization (WHO) and the US Centers for Disease Control and Prevention (CDC) classify extended-spectrum β-lactamase (ESBL)–producing Enterobacteriaceae as a critical or serious threat, underscoring the urgency for enhanced surveillance, antimicrobial stewardship, and infection control strategies [1,2]. In emergency departments (EDs), where patient turnover is rapid and empiric antibiotic use is frequent, the prevalence of multidrug-resistant organisms (MDROs) has risen in recent years, with reported bloodstream infection rates approaching 50% in some settings [3,4]. Established risk factors include advanced age, prior healthcare exposure, and comorbidities [5]; however, most epidemiologic data come from inpatient wards or intensive care units (ICUs), leaving ED-specific trends underexplored, particularly for first-time ED patients without prior medical histories available for reference [6].

Rapid and accurate identification of multidrug-resistant pathogens is essential for effective antimicrobial stewardship and patient management. However, conventional microbiological workflows require bacterial isolation, identification, and antimicrobial susceptibility testing (AST), which may delay definitive microbiological diagnosis and targeted antimicrobial therapy. Recent advances in bioinformatic tools have facilitated the prediction of antimicrobial susceptibility, enabling earlier and more appropriate antimicrobial therapy for critically ill patients and potentially improving clinical outcomes [7]. Whole-genome sequencing (WGS) has demonstrated high accuracy in predicting antimicrobial susceptibility; however, its widespread clinical application remains limited by high cost and resource requirements [8]. Shotgun metagenomic sequencing provides the highest resolution for characterizing microbial communities and antimicrobial resistance determinants but requires substantial computational resources and complex bioinformatic analysis, limiting its routine clinical implementation [9]. In contrast, 16S rRNA sequencing is a more cost-effective and high-throughput approach; however, its lower taxonomic resolution and inability to reliably distinguish certain bacterial species restrict its clinical utility [10]. Despite recent advances in rapid diagnostic technologies, including phenotypic antimicrobial susceptibility testing and other rapid diagnostic platforms, definitive microbiological results are often not immediately available. Consequently, empiric broad-spectrum antibiotics are frequently prescribed, potentially contributing to the emergence and spread of antimicrobial resistance (AMR) [11–14].

The coronavirus disease 2019 (COVID-19) pandemic disrupted healthcare delivery worldwide, altering patient profiles, infection prevention practices, and antimicrobial prescribing patterns [3,15,16]. In Taiwan, the Omicron variant surge in 2022 exhibited three distinct epidemiologic phases, pre-peak stability (January–April), epidemic peak (May–August), and post-peak decline (September–January), as defined by national surveillance data [17]. These stages were marked by resource shortages, increased empiric antibiotic use, and shifting infection control priorities [3,16]. Internationally, surveillance reports and systematic reviews have shown heterogeneous MDRO trends during the pandemic, with some settings reporting increases and others declines, reflecting complex and context-dependent effects of large-scale public health events on antimicrobial resistance [18,19]. For example, an ECDC surveillance report found that while several European countries experienced increases in carbapenem-resistant *Klebsiella pneumoniae* and ESBL-producing *Escherichia coli* during 2020–2021, others reported stable or even decreased rates, likely due to differences in infection prevention measures and antimicrobial prescribing [19]. Similarly, an Italian systematic review demonstrated marked regional variation, with certain hospitals reporting substantial MDRO surges during COVID-19 surges, whereas others maintained or reduced incidence through intensified infection control [18]. Notably, ESBL-producing *K. pneumoniae* has remained a consistently prevalent pathogen in multiple countries during the pandemic [18,20], yet whether similar patterns occurred in ED populations, or evolved across pandemic stages, remains unclear.

SARS-CoV-2 infection has been linked to increased antibiotic consumption, prolonged hospitalization, and higher rates of secondary bacterial infections [21]. Patients with COVID-19 who develop nosocomial bacterial infections, particularly in ICUs, have markedly higher mortality rates (up to 36%) than those without secondary infections [21]. Increases in bloodstream infections caused by ESBL-producing *K. pneumoniae* and *E. coli*, alongside rising resistance to cephalosporins, fluoroquinolones, and carbapenems, have been reported during the pandemic [18,22]. However, evidence suggests that major public health crises can alter not only MDRO prevalence but also the relative importance of specific risk factors over time [23]. Static risk prediction models may underperform when underlying epidemiologic and healthcare dynamics shift, underscoring the need for dynamic, stage-specific recalibration of risk assessment tools [24].

We therefore conducted a retrospective cohort study to investigate the prevalence, resistance patterns, and evolving risk factors for MDRE infections among ED patients during the COVID-19 Omicron wave in Taiwan, across three epidemiologically defined stages. Using an exposome-wide association approach with stage-specific analyses, we hypothesized that (1) MDRE prevalence and resistance profiles would differ across pandemic stages, and (2) the relative importance of key risk factors would shift over time, highlighting the necessity for adaptive antimicrobial stewardship and infection control strategies in acute care settings.

## Materials and methods

### Study population

This retrospective cohort study was conducted in the ED of Chi Mei Medical Center, Liouying Branch, a 900-bed tertiary care hospital in Tainan, Taiwan, between January 1, 2022, and January 31, 2023. Although this was a single-center study, the ED patient flow, diagnostic protocols, and antibiotic stewardship policies at our institution are comparable to those of other tertiary care hospitals in Asia, supporting the external validity of our findings. The study period was divided into three distinct epidemiologic phases, defined according to daily confirmed COVID-19 case counts reported by the Taiwan Centers for Disease Control: a pre-peak stability period (January–April 2022), an epidemic peak dominated by the Omicron variant (May–August 2022), and a post-peak decline (September 2022–January 2023). This phase classification aligns with local CDC surveillance data (https://covid19.mohw.gov.tw/ch/sp-timeline0-205.html, accessed on 16/04/2026) and can be adapted for similar analyses in other healthcare settings. Eligible participants were adult patients (aged ≥20 years) presenting to the ED during the study period who had a blood culture positive for Enterobacteriaceae and complete antimicrobial susceptibility testing (AST) results available. To avoid duplication, only the first positive isolate per patient was included during study period. Patients were excluded if they were younger than 20 years, had mixed infections involving more than one bacterial species, yielded non–Gram-negative isolates, or had missing essential demographic, microbiologic, or laboratory data.

MDRE was defined as Enterobacteriaceae resistant to at least one agent in three or more antimicrobial categories, and CRE was defined as resistance to at least one carbapenem (imipenem, meropenem, or ertapenem), according to Clinical and Laboratory Standards Institute M100 interpretive criteria. Species identification and AST were performed using the VITEK® 2 automated system (bioMérieux, Marcy-l’Étoile, France), with routine quality control using American Type Culture Collection reference strains. Laboratory procedures followed standardized protocols throughout the study period to minimize measurement bias. COVID-19 status was determined by a documented positive SARS-CoV-2 PCR or antigen test result at the time of ED presentation.

Demographic and clinical variables, including age, sex, pandemic phase, bacterial species, AST results, and selected laboratory parameters relevant to infection risk stratification (e.g., white blood cell count, hemoglobin, hematocrit, platelet count, creatinine, alanine aminotransferase, C-reactive protein), were extracted from the hospital’s electronic medical records and microbiology laboratory information system. A complete list of laboratory parameters is provided in S1 Table. Age was analyzed both as a continuous variable and in clinically relevant categories, with ≥75 years defined as “middle elder” based on prior epidemiologic studies linking this threshold to increased MDRO risk (Table 1). To address missing data and minimize bias, we applied multiple imputation by chained equations in R [25], using predictive mean matching for continuous variables and logistic regression for binary variables. The imputation process iteratively modelled each variable with missing values as an outcome predicted by all other variables, cycling until all missing values were replaced. The proportion of missing data was low, and no evidence of systematic association with MDRE status was observed before imputation.

**Table 1:** Basic demographic characteristics and distribution description of patients admitted to Chi Mei Medical Center, Liouying, Tainan, with GNB infections (*N* = 816).

Characteristics, n (%)	Non-MDRE *N* = 529 (64.83)	MDRE *N* = 287 (35.17)	*p*-value
Sex (%)			0.8277
Female	295 (55.77)	157 (54.7)	
Male	234 (44.23)	130 (45.3)	
Age, mean (SD)	72.21 ± 14.25	74.53 ± 13.99	**0.0259**
Age group			0.0788
Age_g (<65 years old)	151 (28.54)	63 (21.95)	
Age_g 1 (65–75 years old)	137 (25.9)	71 (24.74)	
Age_g 2 (75–85 years old)	132 (24.95)	75 (26.13)	
Age_g 3 (≥85 years old)	109 (20.6)	78 (27.18)	
Middle elder (%)	241 (45.56)	153 (53.31)	**0.0411**
Three time points (%)			0.3935
Stage I of the epidemic (4 Months) (2022/01-2022/04)	138 (26.09)	84 (29.27)	
Stage II of the epidemic (4 Months) (2022/05-2022/08)	163 (30.81)	93 (32.4)	
Stage III of the epidemic (5 Months) (2022/09-2023/01)	228 (43.1)	110 (38.33)	

* “Middle elder” is defined as a person aged ≥75 years.

### Statistical analysis

Continuous variables were summarized as means ± standard deviation (SD) and compared using Student’s t-test or the Mann–Whitney U test, as appropriate. Categorical variables were summarized as counts (percentages) and compared using the χ² test or Fisher’s exact test. To identify risk factors for MDRE infection, we employed an exposome-wide association study (ExWAS) approach with logistic regression for each variable, adjusting for potential confounders selected a priori based on clinical relevance and prior literature. Variables with a variance inflation factor >10 were excluded to address multicollinearity. Missing data were handled through complete-case analysis after confirming that the proportion of missing values was low and not systematically associated with MDRE status.

Antimicrobial resistance prevalence was calculated for each species and drug. The five antibiotics with the highest resistance rates were identified and examined in relation to bacterial species across the three pandemic phases. Analyses were performed using R software (version 4.2.2; R Foundation for Statistical Computing, Vienna, Austria) and RStudio (version 2022.12). Two-sided p values <0.05 were considered statistically significant.

## Results

### Descriptive statistics

**Fig 1:**
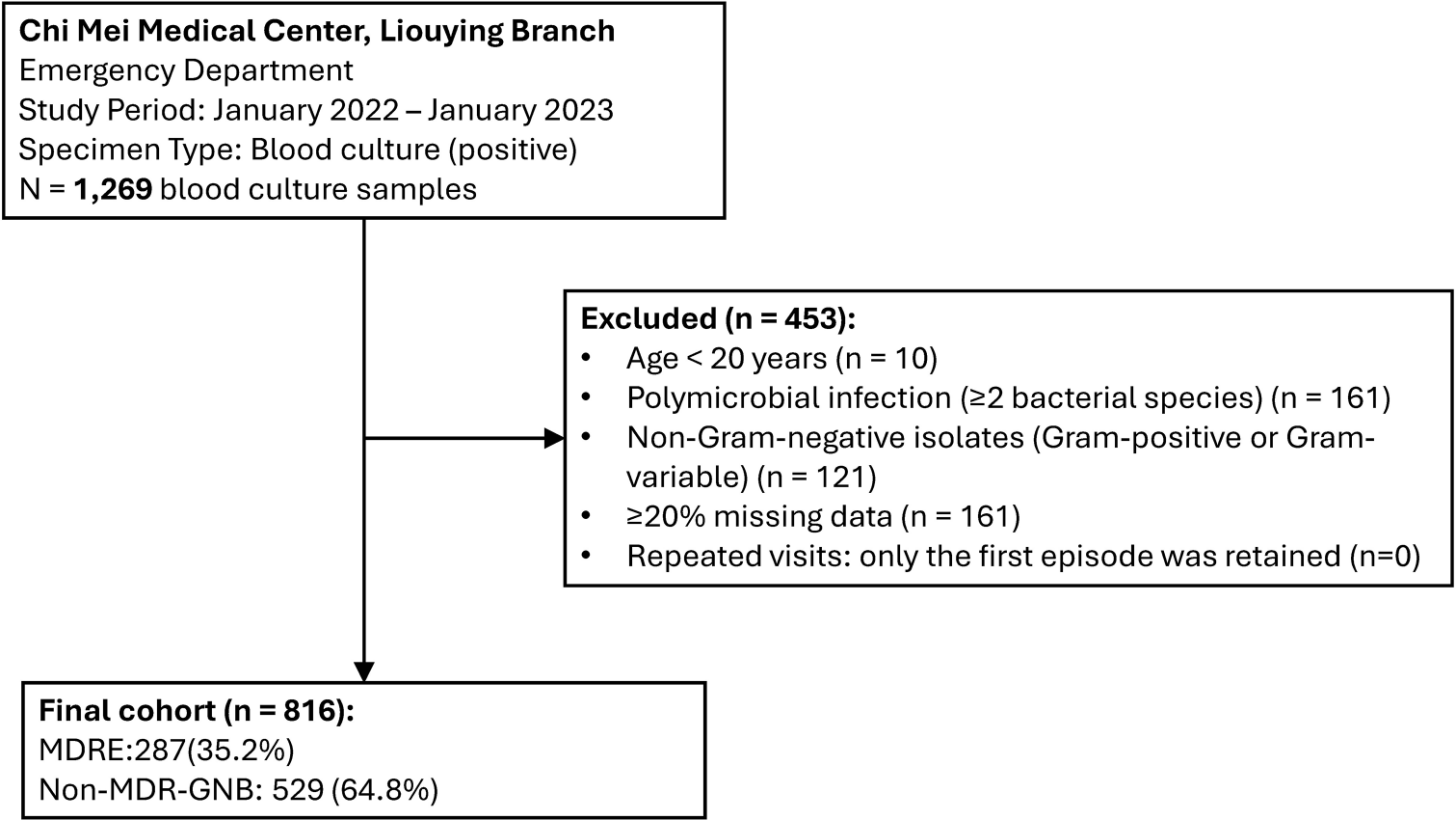
Study flowchart of patient selection and final cohort composition. Abbreviations: ED, emergency department; MDRE, multidrug-resistant Enterobacteriaceae; GNB, Gram-negative bacilli.

From January 2022 to January 2023, a total of 1,269 ED patient records with positive blood cultures were screened. After excluding patients aged < 20 years, those with polymicrobial infections (≥ 2 bacterial species), isolates other than Gram-negative bacilli (GNB), and those with ≥ 20% missing data, 816 unique patients remained for analysis (Fig 1). Of these, 287 (35.2%) had MDRE and 529 (64.8%) had non-MDRE isolates. The mean age in the MDRE group was significantly higher than in the non-MDRE group (74.53 ± 13.99 vs. 72.21 ± 14.25 years, p = 0.026). Patients aged ≥ 75 years (“middle elder”) were disproportionately represented among MDRE cases (p = 0.041). No significant differences were observed in sex distribution or pandemic stage between the two groups (Table 2).

**Table 2:** Comparison of the mean values of various variables before, during, and after the COVID-19 pandemic.

Variables (Mean ± SD)	Stage I of the epidemic	Stage II of the epidemic	Stage III of the epidemic	*p*-value
**Demographic variables**				
Age	72.27 ± 14.73	73.01 ± 14.91	73.54 ± 13.27	0.5825
**Blood glucose**				
Glucose	203.68 ± 135.39	189.87 ± 136.42	200.39 ± 154.28	0.5336
**Kidney function related variables**				
Na	134.14 ± 6.43	133.79 ± 7.81	133.35 ± 6.53	0.4123
K	3.94 ± 0.73	3.92 ± 0.72	3.94 ± 0.75	0.9736
Creatinine	1.53 ± 1.27	1.87 ± 4.16	1.65 ± 1.6	0.3583
**Liver function related variables**				
ALT(SGPT)	55.54 ± 110.32	57.2 ± 109.68	56.55 ± 157.24	0.9904
**Hematology related variables**				
RBC	3.95 ± 0.87	3.85 ± 0.87	4.11 ± 2.31	0.1603
Hb	11.59 ± 2.38	11.69 ± 6.02	11.59 ± 2.13	0.9455
Hct	36.2 ± 23.31	34.53 ± 6.53	34.87 ± 5.97	0.3480
MCV	89.14 ± 9.2	90.87 ± 10.21	89.55 ± 8.99	0.1030
MCH	29.64 ± 3.56	29.86 ± 3.82	29.66 ± 3.7	0.7570
MCHC	33.16 ± 1.57	32.83 ± 1.52	33.09 ± 2.21	0.1067
RDW	14.69 ± 2.47	15.04 ± 2.49	14.55 ± 2.37	0.0503
NRBC	0.09 ± 0.26	0.38 ± 1.85	1 ± 11.43	0.3289
WBC	13.34 ± 6.91	13.24 ± 9.94	12.13 ± 6.61	0.1173
Platelet	211.61 ± 118.08	195.65 ± 94.31	198.87 ± 107.52	0.2275
**Inflammation related marker**				
Band	0.8 ± 2.92	0.88 ± 2.95	0.69 ± 2.31	0.6889
Segment	89.12 ± 50.12	87.81 ± 51.25	85.15 ± 12.32	0.4784
Lymphocyte	8.18 ± 9.62	8.35 ± 9.6	8.23 ± 8.83	0.9787
Monocyte	4.27 ± 4.23	4.13 ± 3.42	4.62 ± 5.9	0.4382
Eosinophil	0.33 ± 0.56	0.61 ± 1.66	0.7 ± 2.9	0.1293
Basophil	0.19 ± 0.26	0.24 ± 1.01	0.25 ± 0.97	0.7060
CRP/hs CRP	123.34 ± 103.58	117.42 ± 102.83	115.48 ± 96.58	0.6554

A p < 0.05 was considered statistically significant. Abbreviations: CRP: C-reactive protein; Hb, hemoglobin; Hct, hematocrit; hs-CRP = high-sensitivity CRP; MCV = mean corpuscular volume; MCH = mean corpuscular hemoglobin; MCHC = mean corpuscular hemoglobin concentration; NRBC = nucleated RBC; RBC, red blood cells; RDW, red cell distribution width; WBC, white blood cell count.

### Stage-specific analysis of laboratory parameters in MDRE and non-MDRE groups

**Fig 2:**
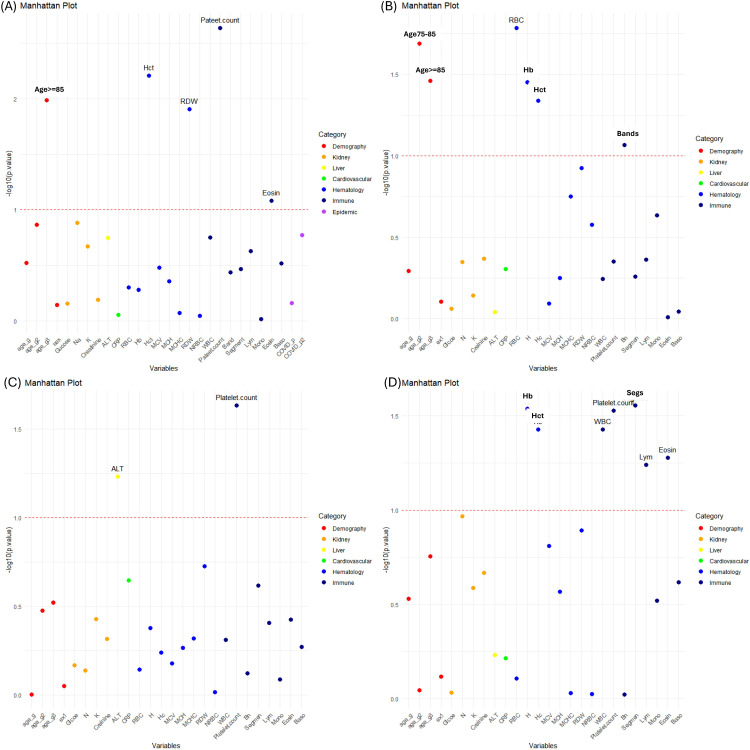
Stage-specific associations between clinical variables and MDRE infection identified by exposome-wide association analysis. Manhattan plots showing variables significantly associated with MDRE infection in ED patients across COVID-19 Omicron pandemic phases in Taiwan: (A) all stages combined, (B) Stage I (pre-peak), (C) Stage II (epidemic peak), and (D) Stage III (post-peak). The horizontal dashed line indicates the significance threshold (p < 0.05). Variables are color-coded by clinical category. Pandemic stage definitions were based on national case counts from the Taiwan Centers for Disease Control. Abbreviations: ALT, alanine aminotransferase; Bands, band neutrophils; Eosin, eosinophils; Hb, hemoglobin; Hct, hematocrit; Lym, lymphocytes; Na, sodium; RBC, red blood cells; RDW, red cell distribution width; Segs, segmented neutrophils; WBC, white blood cell count.

Using national COVID-19 surveillance data from the Taiwan Centers for Disease Control (case counts reflect the national epidemic curve, not the study cohort), the 2022 Omicron wave was divided into Stage I (pre-peak, January–April; 133,368 reported cases), Stage II (epidemic peak, May–August; 6,330,945 cases), and Stage III (post-peak decline, September 2022–January 2023; 2,703,498 cases). When all stages were combined, older age, hematocrit (Hct), red cell distribution width (RDW), eosinophil count, and platelet count were associated with MDRE infection (all p < 0.05; Fig 2A). Stage-specific analyses revealed distinct patterns. In Stage I, variables associated with MDRE infection included the “middle elder” age group (≥ 75 years), red blood cell (RBC) count, hemoglobin (Hb), and Hct (all p < 0.05; Fig 2B). Odds ratio (OR) analysis indicated higher odds for patients aged 75–85 years (OR = 2.48, 95% CI: 1.16–5.43) and ≥ 85 years (OR = 2.29, 95% CI: 1.07–5.00) compared with younger groups, suggesting that demographic variables had a stronger association than hematologic parameters in the early pandemic stage. In Stage II, platelet count and alanine aminotransferase (ALT) were associated with MDRE infection (both p < 0.05; Fig 2C), with ORs of 1.00 (95% CI: 1.00–1.01) and 1.00 (95% CI: 0.99–1.00), respectively. By Stage III, immune-related laboratory variables became more prominent: Hb, Hct, platelet count, white blood cell (WBC) count, and segmented neutrophil count (Segs) were all associated with MDRE infection (p < 0.05; Fig 2D), and OR analysis showed stronger associations for WBC and Segs than for demographic variables.

**Fig 3:**
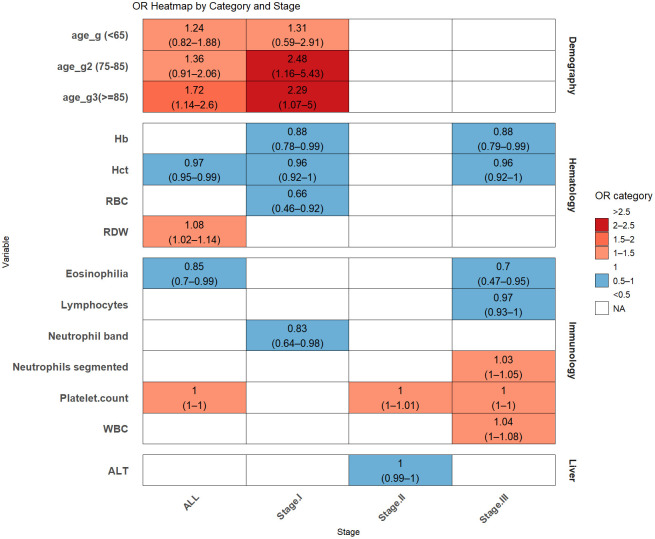
Stage-specific odds ratios for characteristics associated with MDRE infection in ED patients. Heatmap displaying odds ratios (ORs) and 95% confidence intervals (CIs) for variables significantly associated with MDRE infection across the same pandemic phases as in Fig 2. Variables are grouped by clinical category. Red tones indicate OR > 1 (higher odds of MDRE infection), blue tones indicate OR < 1 (lower odds), and white cells indicate no statistically significant association in that stage. Pandemic stage definitions were based on national case counts from the Taiwan Centers for Disease Control.

Taken together, these findings indicate that variables associated with MDRE infection in ED patients shifted across pandemic stages, from demographic dominance in Stage I, to isolated laboratory markers in Stage II, to immune and inflammatory indices in Stage III (Figs 2 and 3). Similar temporal changes in antimicrobial resistance–related variables have been reported internationally during COVID-19 surges, highlighting the need for adaptive, stage-specific risk assessment strategies in acute care environments.

### Stage-dependent trends in antibiotic resistance among predominant MDRE species

Antibiotic resistance profiling identified *E. coli*, *K. pneumoniae*, *Proteus mirabilis*, *Enterobacter cloacae*, and *Citrobacter koseri* as the five most common MDRE species. Across all isolates, the antibiotics with the highest resistance rates were ampicillin (74.6%), cefazolin (44.9%), ciprofloxacin (37.2%), amoxicillin–clavulanate (33.8%), and cefuroxime (28.8%). At the species–antibiotic pair level, *K. pneumoniae* demonstrated universal resistance to ampicillin (142/142 isolates) and 40.1% resistance to ciprofloxacin (57/142). *E. coli* showed resistance to ampicillin in 71.2% of isolates (358/503), cefazolin in 44.7% (225/503), and ciprofloxacin in 42.0% (211/502). Both *E. cloacae* and *C. koseri* exhibited 100% resistance to ampicillin, while *P. mirabilis* and *E. cloacae* showed 100% resistance to cefazolin.

**Fig 4:**
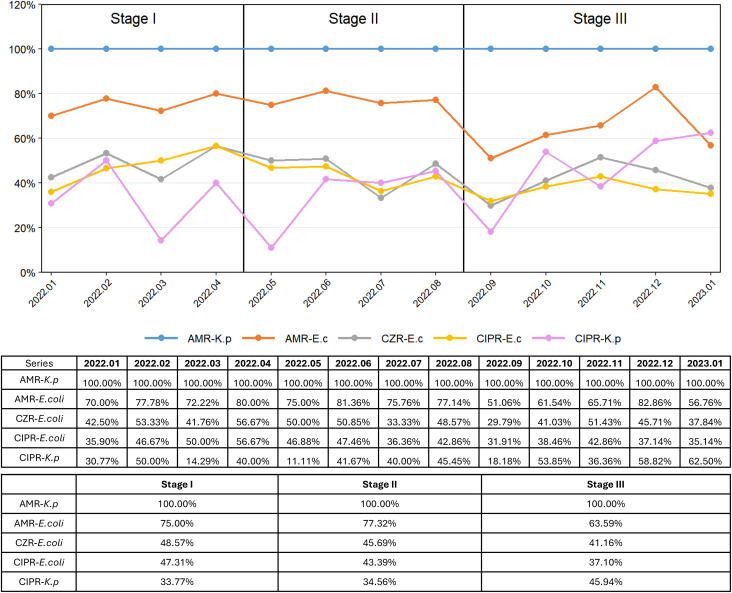
Temporal trends in resistance rates for key bacteria–antibiotic pairs across pandemic stages. Monthly resistance rates are shown for ampicillin-resistant *K. pneumoniae* (AMR-K.p), ampicillin-resistant *E. coli* (AMR-E.c), cefazolin-resistant *E. coli* (CZR-E.c), ciprofloxacin-resistant *E. coli* (CIPR-E.c), and ciprofloxacin-resistant *K. pneumoniae* (CIPR-K.p) from January 2022 to January 2023, stratified by Stage I (pre-peak), Stage II (epidemic peak), and Stage III (post-peak). Ampicillin resistance in *K. pneumoniae* remained universally high (100%) throughout, while ciprofloxacin resistance in *K. pneumoniae* showed a progressive increase. In contrast, resistance rates in *E. coli* generally declined over time for ampicillin, cefazolin, and ciprofloxacin.

Temporal trend analysis (Fig 4) demonstrated stage-dependent patterns. Ampicillin resistance in *K. pneumoniae* remained at 100% throughout the study period, whereas resistance in *E. coli* declined to 63.6% by Stage III. Ciprofloxacin resistance in *K. pneumoniae* increased progressively across all stages, in contrast to *E. coli*, where resistance rates declined over time. These trends are consistent with surveillance data from the European Centre for Disease Prevention and Control and reports from Italy, where pandemic peaks coincided with increased *K. pneumoniae* fluoroquinolone resistance despite overall reductions in certain MDRO rates—likely reflecting selective pressure from empiric fluoroquinolone use in COVID-19 patients, particularly in intensive care and other high-acuity settings.

## Discussion

This study demonstrates that the clinical and laboratory variables associated with MDRE infection in ED patients shifted markedly across the pandemic phases of the COVID-19 Omicron wave. In the pre-peak stage, older age (≥75 years) and hematologic indices were most salient; during the epidemic peak, platelet count and ALT emerged; and in the post-peak period, immune and inflammatory markers became more prominent. In parallel, *K. pneumoniae* maintained universal ampicillin resistance with a progressive rise in ciprofloxacin resistance, whereas *E. coli* showed declining resistance to multiple agents. These temporally dynamic patterns suggest that static risk assessment frameworks may lose predictive accuracy during major healthcare disruptions, and that incorporating stage-specific markers into ED-based empirical therapy algorithms could optimize antimicrobial use and improve patient outcomes.

To characterize these temporal changes, we applied a stage-based analytic framework to MDRE epidemiology in ED patients during the COVID-19 Omicron wave in Taiwan, referencing national surveillance data from the Taiwan Centers for Disease Control and the pandemic phase definitions proposed by Lai et al. The 13-month study period (January 2022–January 2023) was divided into three phases: pre-peak, epidemic peak, and post-peak. Among 1,269 positive ED blood cultures screened, 816 cases met eligibility criteria after exclusions. Using an ExWAS approach, we found that the relevance of demographic and laboratory variables shifted by phase: older age and red blood cell indices dominated in the pre-peak stage; platelet count and ALT were most relevant during the peak; and immune cell counts and platelet count dominated post-peak. Odds ratio heatmaps confirmed these shifts, with older age losing significance after the early stage, while platelet count and immune markers gained prominence. Similar temporal variability has been reported internationally, with studies in Uganda and China identifying older age as a consistent MDRO-associated factor [26,27], and European and Italian surveillance showing pathogen-specific resistance shifts during COVID-19 surges [18,19]. Our findings extend these observations by demonstrating phase-dependent changes within a single pandemic wave, suggesting that static risk models may lose predictive accuracy during major healthcare disruptions [23,24].

Several mechanisms may explain these shifts. Early in the pandemic, older adults’ vulnerability likely reflected immunosenescence, multimorbidity, and repeated healthcare exposure [26,27]. During the peak, platelet count and ALT may have been influenced by COVID-19 related hematologic effects (platelet activation, immune-mediated consumption) and liver injury from viral infection or systemic inflammation [28,29]. By the post-peak stage, immune cell counts (WBC, Segs) became more prominent, potentially reflecting changes in patient mix, severity of bacterial infections, or hospital triage patterns as COVID-19 case burdens eased.

Antibiotic resistance profiling identified *E. coli*, *K. pneumoniae*, *P. mirabilis*, *E. cloacae*, and *C. koseri* as the predominant MDRE species. Across all isolates, resistance was highest to ampicillin (74.6%), cefazolin (44.9%), ciprofloxacin (37.2%), amoxicillin–clavulanate (33.8%), and cefuroxime (28.8%). K. pneumoniae maintained 100% ampicillin resistance throughout and showed steadily rising ciprofloxacin resistance. In contrast, *E. coli* resistance to ampicillin declined to 63.6% by Stage III, with declines also in cefazolin and ciprofloxacin resistance. These patterns parallel ECDC surveillance [19] and Italian reports [18], which noted increased *K. pneumoniae* fluoroquinolone resistance during pandemic peaks despite reductions in other MDROs. The persistence of ciprofloxacin resistance in *K. pneumoniae* may reflect high empiric use during COVID-19 to manage suspected bacterial co-infections, particularly in ICU and high-acuity settings [30–33], whereas declines in *E. coli* resistance may relate to altered prescribing or reduced healthcare exposure in some populations. The observed 100% ampicillin resistance in K. pneumoniae is consistent with its well-recognized intrinsic resistance to ampicillin, which is primarily mediated by chromosomally encoded SHV β-lactamase [34]. Ampicillin was nevertheless retained in the analysis because susceptibility data were consistently available across all study stages, enabling longitudinal comparisons of resistance patterns.

These findings underscore that major public health events can shift the relevance of established MDRE-associated variables, highlighting the need for dynamic recalibration of risk models and context-specific antimicrobial stewardship strategies. WHO, CDC, and ECDC now emphasize that AMR surveillance should be dynamic and situational [19,24]. In ED practice, this could mean incorporating stage-adapted variables, such as platelet count during epidemic peaks or immune markers in post-peak phases, into triage algorithms for empirical therapy and infection control planning. This framework could be adapted globally to optimize antimicrobial use and resource allocation during pandemics or other disruptive events (e.g., SARS, MERS, natural disasters).

Our study is limited by its single-center design, which may affect generalizability; however, our ED’s patient flow, diagnostic protocols, and stewardship policies are comparable to other tertiary hospitals in Asia. The sample size in some subgroups was small, limiting statistical power, and available laboratory data were restricted to routine ED tests without detailed information on prior hospitalization, healthcare exposure, antibiotic exposure, or comorbidity information. The observational design also precludes causal inference.

In conclusion, variables associated with MDRE infection and species-specific resistance trends shifted substantially over a single pandemic wave, likely reflecting evolving patient demographics, infection severity, and healthcare pressures. Recognizing and integrating such temporal variability into AMR surveillance and clinical decision-making can improve the precision of empirical antibiotic use and strengthen infection control preparedness for future public health emergencies. This stage-adapted approach could be implemented globally to optimize antimicrobial stewardship and infection control strategies during future pandemics or other disruptive public health events.

## Supporting information

S1 Table Demographic and laboratory variables. (DOCX)
