# Supplementary material for: Changes in multidrug-resistant Enterobacteriaceae features among patients in the emergency room during the COVID-19 Omicron variant pandemic
Source: PLoS One. 2026 Aug 17;21(8):e0355725. doi: 10.1371/journal.pone.0355725 (PMC13480630; doi:10.1371/journal.pone.0355725)
Supplement: Supplementary file 1 [file pone.0355725.s001.docx]

**Supplementary Table S1.** Demographic and laboratory variables.

This table summarizes the demographic characteristics and laboratory parameters included in the analysis. Missing data were addressed using complete-case analysis after confirming that the proportion of missing values was low and not systematically associated with MDRE status, thereby minimizing the risk of bias.

| **Category** | **Variable (Abbreviation)** | **Description** | **Unit** |
| --- | --- | --- | --- |
| Demographics | Age | Age in years at index date | years |
| Demographics | Sex | Biological sex (male/female) | — |
| Laboratory data | Glucose | Serum glucose concentration | mg/dL |
| Laboratory data | Sodium (Na) | Serum sodium concentration | mmol/L |
| Laboratory data | Potassium (K) | Serum potassium concentration | mmol/L |
| Laboratory data | High-sensitivity Troponin I (hs-Troponin I) | Serum high-sensitivity troponin I level | ng/L |
| Laboratory data | Blood urea nitrogen (BUN) | Serum blood urea nitrogen level | mg/dL |
| Laboratory data | Creatinine | Serum creatinine level | mg/dL |
| Laboratory data | Aspartate aminotransferase (AST) | Serum AST level | U/L |
| Laboratory data | Alanine aminotransferase (ALT) | Serum ALT level | U/L |
| Laboratory data | High-sensitivity C-reactive protein (hs-CRP) | Serum hs-CRP level | mg/L |
| Laboratory data | Albumin | Serum albumin concentration | g/dL |
| Laboratory data | Total protein | Serum total protein concentration | g/dL |
| Laboratory data | Red blood cell count (RBC) | RBC count | ×10^6/µL |
| Laboratory data | Hemoglobin (Hb) | Hemoglobin concentration | g/dL |
| Laboratory data | Hematocrit (Hct) | Percentage of blood volume occupied by red blood cells | % |
| Laboratory data | Mean corpuscular volume (MCV) | Average volume of red blood cells | fL |
| Laboratory data | Mean corpuscular hemoglobin (MCH) | Average hemoglobin content per red blood cell | pg |
| Laboratory data | Mean corpuscular hemoglobin concentration (MCHC) | Average hemoglobin concentration in red blood cells | g/dL |
| Laboratory data | Red blood cell distribution width (RDW) | Variation in red blood cell size | % |
| Laboratory data | White blood cell count (WBC) | WBC count | ×10^3/µL |
| Laboratory data | Platelet count | Platelet count | ×10^3/µL |
| Laboratory data | Nucleated red blood cell count (NRBC) | NRBC count | ×10^3/µL |
| Laboratory data | Neutrophil band | Percentage of band neutrophils | % |
| Laboratory data | Segmented neutrophils | Percentage of segmented neutrophils | % |
| Laboratory data | Lymphocytes | Percentage or count of lymphocytes | % or ×10^3/µL |
| Laboratory data | Monocytes | Percentage or count of monocytes | % or ×10^3/µL |
| Laboratory data | Eosinophils | Percentage or count of eosinophils | % or ×10^3/µL |
| Laboratory data | Basophils | Percentage or count of basophils | % or ×10^3/µL |
| Other | COVID-19 | COVID-19 infection status (positive/negative) | — |
